# *In Vivo* Bone Regeneration Capacity of Multiscale Porous Polycaprolactone-Based High Internal Phase Emulsion (PolyHIPE) Scaffolds in a Rat Calvarial Defect Model

**DOI:** 10.1021/acsami.3c04362

**Published:** 2023-05-30

**Authors:** Betül Aldemir Dikici, Min-Chia Chen, Serkan Dikici, Hsien-Chung Chiu, Frederik Claeyssens

**Affiliations:** † Department of Bioengineering, Izmir Institute of Technology, Urla, Izmir 35433, Turkey; ‡ Department of Periodontology, School of Dentistry, National Defense Medical Center and Tri-Service General Hospital, Taipei 114, Taiwan, ROC; § Private Dental Clinic of New Taipei City, Taipei 220, Taiwan, ROC; ∥ Kroto Research Institute, Department of Materials Science and Engineering, University of Sheffield, Sheffield S37HQ, United Kingdom; ⊥ INSIGNEO Institute for In Silico Medicine, Department of Materials Science and Engineering, University of Sheffield, Sheffield S13JD, United Kingdom

**Keywords:** polycaprolactone, emulsion templating, 3D printing, stereolithography, bone tissue engineering, multiscale porosity, *in vivo*, rat calvarial defect

## Abstract

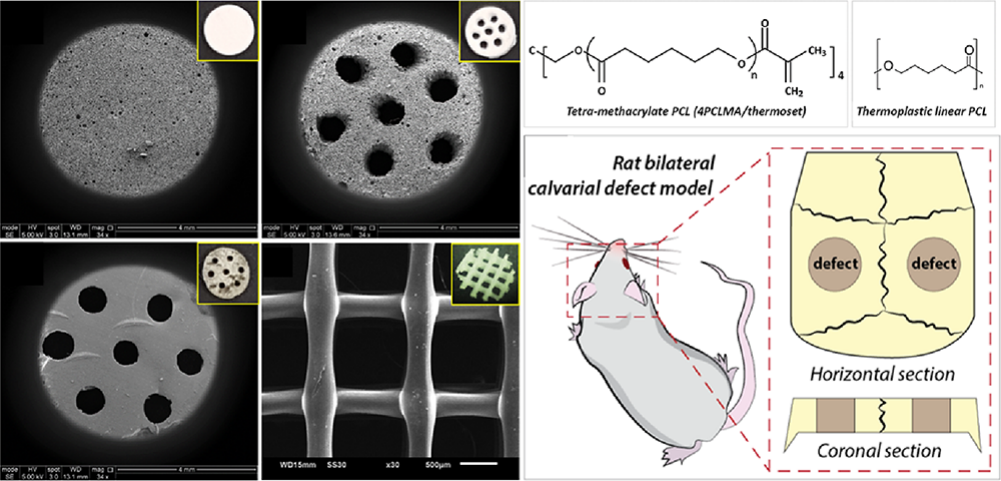

Globally, one of the most common tissue transplantation procedures is bone grafting. Lately, we have reported the development of polymerized high internal phase emulsions (PolyHIPEs) made of photocurable polycaprolactone (4PCLMA) and shown their potential to be used as bone tissue engineering scaffolds *in vitro*. However, it is essential to evaluate the *in vivo* performance of these scaffolds to investigate their potential in a clinically more relevant manner. Therefore, in this study, we aimed to compare *in vivo* performances of macroporous (fabricated using stereolithography), microporous (fabricated using emulsion templating), and multiscale porous (fabricated using emulsion templating and perforation) scaffolds made of 4PCLMA. Also, 3D-printed macroporous scaffolds (fabricated using fused deposition modeling) made of thermoplastic polycaprolactone were used as a control. Scaffolds were implanted into a critical-sized calvarial defect, animals were sacrificed 4 or 8 weeks after implantation, and the new bone formation was assessed by micro-computed tomography, dental radiography, and histology. Multiscale porous scaffolds that include both micro- and macropores resulted in higher bone regeneration in the defect area compared to only macroporous or only microporous scaffolds. When one-grade porous scaffolds were compared, microporous scaffolds showed better performance than macroporous scaffolds in terms of mineralized bone volume and tissue regeneration. Micro-CT results revealed that while bone volume/tissue volume (Bv/Tv) values were 8 and 17% at weeks 4 and 8 for macroporous scaffolds, they were significantly higher for microporous scaffolds, with values of 26 and 33%, respectively. Taken together, the results reported in this study showed the potential application of multiscale PolyHIPE scaffolds, in particular, as a promising material for bone regeneration.

## 1 Introduction

Critical-sized bone defects due to infections, fractures, tumor resection, and osteoporosis cannot heal by themselves within the host tissue and remain a clinical problem worldwide. Allografts and autografts have been widely used for the regeneration of large defects; however, they have disadvantages, such as the need for further processes to be able to prevent disease transmission and limited availability, respectively.^1^ To overcome these shortcomings, researchers have developed bone graft substitutes made of biomaterials using various fabrication techniques to create porous substrates and provide a space for newly formed tissue. Electrospinning,^2,3^ 3D printing,^1,4^ porogen leaching,^5,6^ and gas foaming^7,8^ are some of the most widely used routes for the development of bone tissue engineering scaffolds.

**Figure 1:**
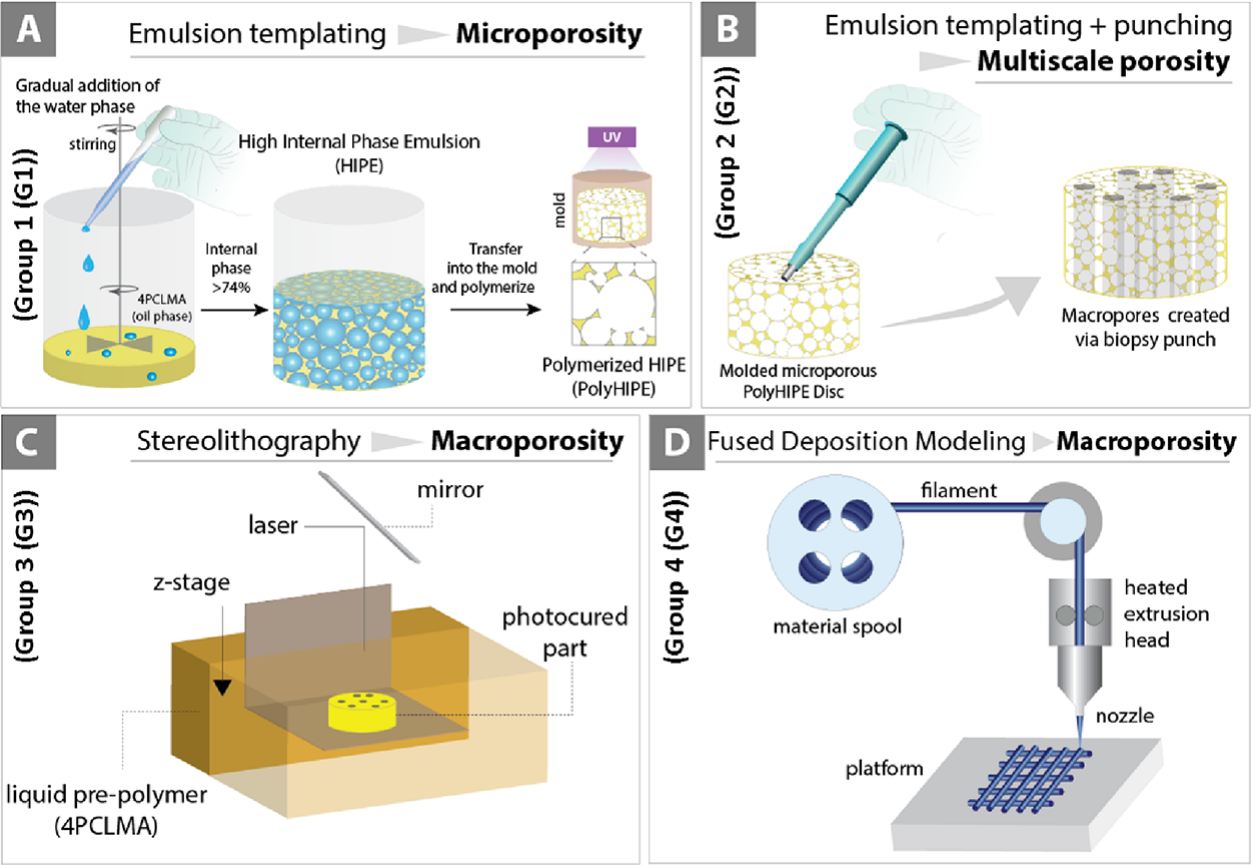
(A) PolyHIPEs were fabricated via emulsion templating (group 1 (G1)), (B) scaffolds with multiscale porosity were fabricated by combining emulsion templating and perforation (group 2 (G2)), (C) 4PCLM-based macroporous scaffolds were fabricated via stereolithography (group 3 (G3)), and (D) thermoplastic PCL-based macroporous scaffolds were created via fused deposition modeling (group 4 (G4)).

In the past decade, the emulsion templating method also attracted attention due to its various advantages, such as enabling the manufacturing of scaffolds with high porosity and interconnectivity, high tunability of morphological features, and fabrication of complex structures by being suitable to be combined with other fabrication techniques.^1,9−13^ In this technique, a biphasic emulsion is created, and the continuous phase is polymerized. Droplets of the internal phase behave as pore templates during polymerization (Figure 1A). Emulsions with dispersed droplet phase (φ) greater than 74.05% are categorized as a high internal phase emulsion (HIPE), and the substrates that are created from their polymerization are defined as polymerized HIPE (PolyHIPE).

Polycaprolactone (PCL) is one of the most widely preferred synthetic biomaterials for the development of bone grafts and scaffolds because of its advantages, such as being biodegradable and bioresorbable and the existence of PCL-based Food and Drug Administration (FDA)-approved medical devices in the clinics.^14^ However, although PolyHIPEs made of various biodegradable polymers such as fumarate^15−18^ and thiolenes^19−22^ have been previously reported, the development of PCL-based PolyHIPEs was found challenging due to the high viscosity of PCL that limits the mixing of two phases during emulsification.^21,23−28^

Lately, we have reported the development of fully PCL-based PolyHIPEs^29^ and their potential to be used as bone tissue engineering scaffolds *in vitro.*(1,30) PCL PolyHIPEs provided a suitable matrix for attachment, proliferation, infiltration of the cells, and the formation of the extracellular matrix (ECM) of mouse post-osteoblast/pre-osteocytes,^1,29^ human fibroblasts,^29,31−33^ human mesenchymal progenitors,^1^ and human endothelial cells.^34^ Similar to our findings, the morphology of the PolyHIPE scaffolds made of various biomaterials has been found favorable for bone regeneration *in vitro* by many researchers;^16,35−38^ however, to date, there have been no studies reporting the *in vivo* performance of any PolyHIPEs as bone tissue engineering scaffolds. We previously reported the performance of PolyHIPEs designed as bone tissue engineering scaffolds on chick chorioallantoic membrane (CAM) assay.^1,30^ However, it is essential to evaluate the *in vivo* bone regeneration capacity of these scaffolds to investigate their potential in a clinically more relevant manner.

In this study, our goal was to assess the *in vivo* performance of PCL PolyHIPEs with micropores ranging from 6 to 78 μm in a rat calvarial defect model. We also investigated the effect of micro-, macro-, and multiscale porosity on bone formation *in vivo*. For this, in addition to the microporous PCL PolyHIPE group (fabricated using emulsion templating), macroporous discs were fabricated using micro-stereolithography (Figure 1C), and the multiscale porous group was fabricated by creating macropores on PCL PolyHIPE discs (Figure 1B). The PCL we used for the fabrication of these three groups was tetra-methacrylated PCL (4PCLMA), which is photocurable. As 3D-printed thermoplastic PCL (not photocurable) has been commonly preferred in the manufacturing of bone tissue engineering scaffolds and its *in vivo* performance^39−43^ has been evaluated in the literature, we also have included a control group made of a thermoplastic PCL-based scaffold fabricated using fused deposition modeling (FDM) (Figure 1D). 4 and 8 week post-implantation scaffolds were isolated and analyzed using dental radiography, micro-CT, and histology.

## 2 Experimental Section

### 2.1 Materials

Methanol (MeOH), dichloromethane (DCM), chloroform, and isopropyl alcohol were obtained from Fisher Scientific (Pittsburgh, USA). Pentaerythritol, ε-caprolactone, methacrylic anhydride (MAAn), 2,4,6-trimethylbenzoyl phosphine oxide/2-hydroxy-2-methylpropiophenone blend (photoinitiator), tin(II) 2-ethylhexanoate, β-carotene, triethylamine (TEA), and hydrochloric acid (HCl) were obtained from Sigma-Aldrich (Poole, UK). Hypermer B246 (the surfactant) was provided as a gift by Croda. PCL pellets were purchased from Capa 6500 and used as they were received (Perstorp Holding AB, Sweden).

### 2.2 Synthesis of 4PCLMA

Photocurable PCL was synthesized by following the protocol, which is described in detail elsewhere.^1,29,30,34^ Briefly, ε-caprolactone and pentaerythritol were weighed and mixed at 160 °C in a flask until pentaerythritol was dissolved. Then, tin(II) 2-ethylhexanoate was added, and the system was left for the reaction for 24 h. At the end of the synthesis of the hydroxyl-terminated polymer, the system was cooled down to room temperature (RT). The obtained 4PCL was dissolved in DCM. After TEA addition, the flask was placed in an ice bath. In a separate beaker, MAAn was dissolved in DCM and added via a dropping funnel before the system was kept at RT. Then, 4PCLMA was washed with HCl solution and deionized water, respectively, and the solvent was removed using a rotary evaporator. Finally, the polymer was washed with methanol, and the solvent was removed. The resulting 4PCLMA was stored for further use.

### 2.3 Fabrication of the Scaffolds

#### 2.3.1 Fabrication of G1 and G2 Scaffolds

**Figure 2:**
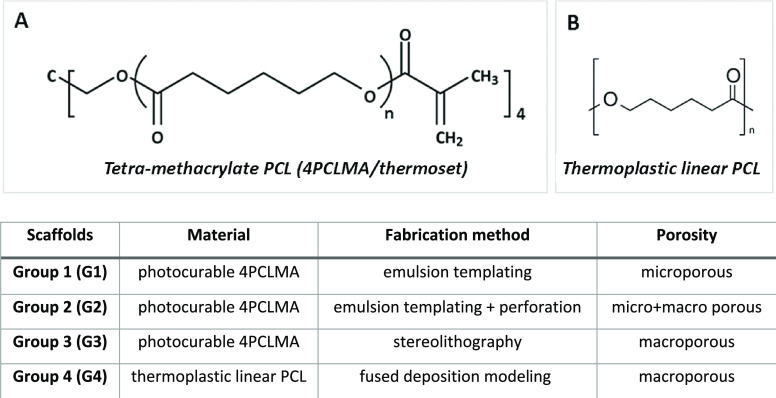
Polymers used in the composition of the scaffolds. (A) 4PCLMA was used for the development of microporous, multiscale porous, and macroporous scaffolds, and (B) thermoplastic PCL was used for the fabrication of 3D-printed macroporous scaffolds. (Table) Scaffolds and their properties.

Four groups of scaffolds were fabricated in the scope of this study: group 1 (G1); microporous, group 2 (G2); multiscale porous, group 3 (G3); macroporous, group 4 (G4); macroporous. Material and scaffold properties are given in more detail in Figure 2.

**Figure 3:**
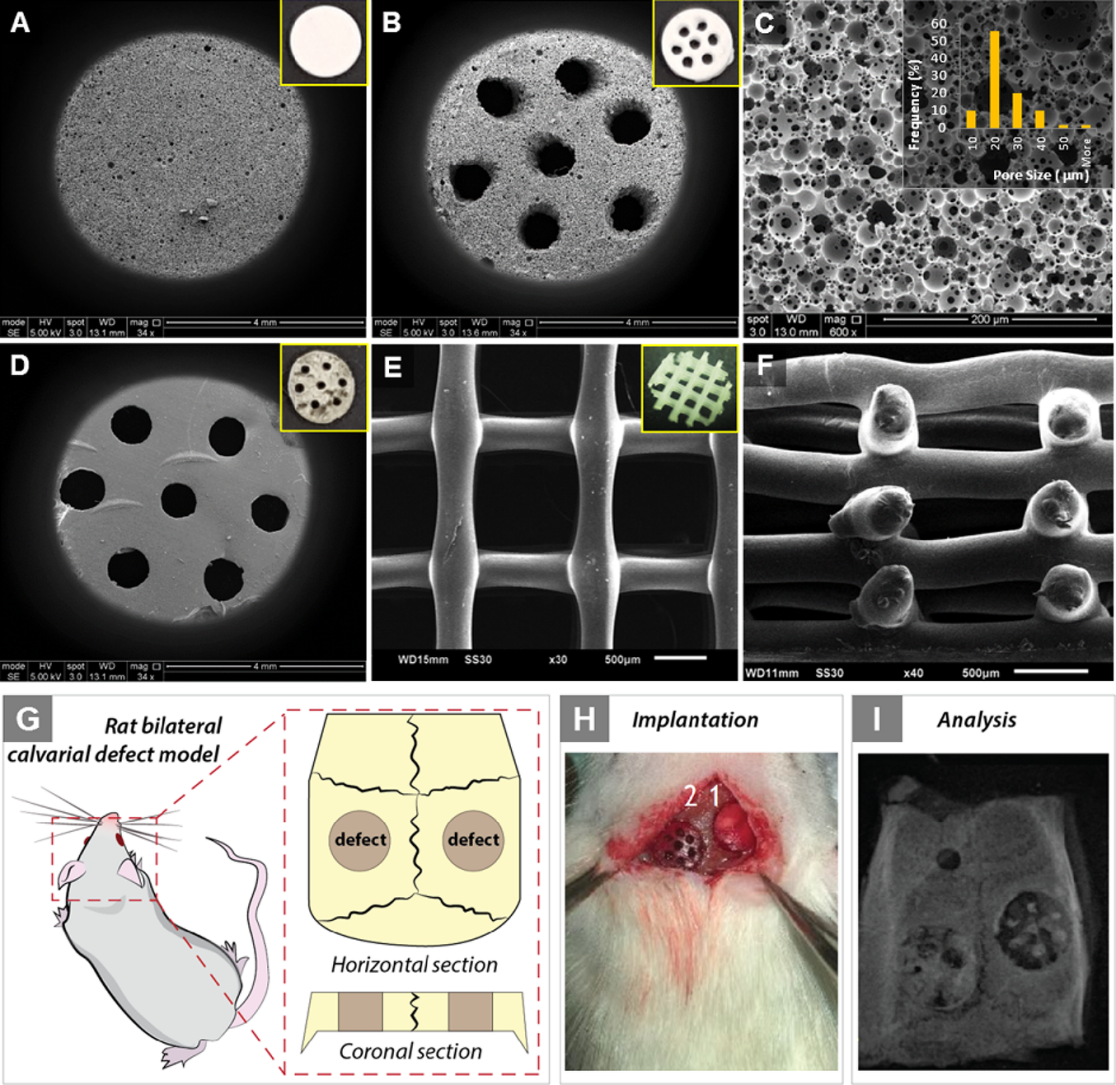
Scanning electron microscopy (SEM) images of (A) G1, (B) G2, (C) the surface of G1 in higher magnification and its micropore size distribution histogram, (D) G3, (E) G4 (top view), and (F) G4 (side view). (G–I) Schematic diagram demonstrating the main experimental procedure in this study. (G) Creating a bilateral defect in rat calvaria, (H) implantation of the scaffolds, and (I) analysis after 4 and 8 weeks of experiments.

For G1 and G2, briefly, 0.4 g of 4PCLMA, 10% (w/w) surfactant, and 0.6 g of chloroform/toluene (80%/20% (w/w)) solvent blend were added to a glass container and mixed at 375 rpm by a magnetic stirrer for 1 min to create a homogeneous mixture.^29,44^ Then, water (as an internal phase, 4 mL) was added to the mixture dropwise, and the emulsion was mixed for further 2 min at 375 rpm. Both sides of the 4PCLMA HIPE were photo-cured in the syringe for 5 min (Omnicure Series 1000, Lumen Dynamics, Canada). The obtained PolyHIPEs were soaked in methanol to wash any remaining contaminants of uncured polymer, Hypermer B246, or solvent. Then, the cylindrical scaffolds were transferred to water and frozen in the freezer and dried in the vacuum oven. PolyHIPEs were sliced into 1.8 mm-thick discs to create G1 scaffolds. G2 scaffolds were fabricated in the same way as with G1, and obtained scaffolds were perforated to create seven macropores (Figure 3).

#### 2.3.2 Fabrication of G3 Scaffolds

G3 scaffolds were fabricated by micro-stereolithography^45−47^ using 4PCLMA that was mixed with a 3.2% (w/w) photoinitiator and 0.05% β-carotene. In this setup, a cross-sectional design of the 3D model was uploaded to the digital micromirror device (DMD) (Texas Instruments Incorporated, USA, associated software: ALP-3 Basic, ViALUX GmbH). The beam was expanded at 10 mW and aligned while running through a mirror set and a spatial filter (Vortran Laser Technology Inc., USA). Once the beam reached the DMD, an uploaded cross-sectional image was reflected. 4PCLMA was placed under a motorized *z*-axis translation stage. The stage moved down into the liquid 4PCLMA with a maximum velocity of 0.01 mm/s, and the irradiated regions were polymerized (Thorlabs Ltd., UK, associated software: APT software). Once the final design was fabricated, the scaffolds were recovered from the stage and washed with isopropyl alcohol to remove the uncured prepolymer and photoinitiator. Then, the same washing and drying procedure of G1 and G2 scaffolds was applied to G3.

#### 2.3.3 Fabrication of G4 Scaffolds

PCL filaments with a diameter of 1.70 ± 0.08 mm were manufactured from PCL beads using an extruder (Filabot, Barre, Milwaukee, WI, USA). Disc-shaped scaffolds with a 6 mm diameter and 1.8 mm thickness were created in CAD software (SolidWorks Corp., Dassault Systemes S.A.), then exported into slicing software (Simplify 3D, US) where the scaffold structure was designed into three lay-down patterns at 0/90° with a 70% porosity setting in the software, and exported into the STL file format. The PCL filament was inserted into a FDM printer to fabricate the scaffolds. The nozzle diameter was 0.4 mm, and the printing speed was 10 mm/s. The fabricated scaffolds were soaked in 70% ethanol overnight and UV-radiated for 4 h before implantation (PHILIPS, Eindhoven, Netherlands).^48^

### 2.4 Scanning Electron Microscopy (SEM)

SEM was used to explore the morphology of the fabricated scaffolds.^9,29,49^ All samples were gold-coated to increase the conductivity. For morphological investigation of G1, G2, and G3, a FEI Inspect F SEM (Philips/FEI XL-20 SEM, Cambridge, UK) was used and, for G4, Jeol SEM was used (JSM-6510, Japan) with 10 kV power. Fifty micropores and 10 macropores were selected randomly, and measurements were taken for the calculation of the average pore sizes. A statistical correction factor (2/√3) was also applied for the measured values to correct the underestimation of the measurements due to uneven sectioning.^50^

### 2.5 *In Vivo* Studies

The operations were conducted in the Laboratory Animal Center of the National Defense Medical Center (NDMC-LAC), Taiwan (approved number: IACUC-17-310 (partial from IACUC-23-323)). All experiments were implemented in accordance with relevant animal experiments guidelines and regulation protocol. NDMC-LAC determined the number of animals, which could prove the efficiency of statistical data and complied with the ARRIVE guidelines. A total of 20, 8 week-old male Sprague–Dawley rats weighing 300–350 g were selected. All animals were housed in a pathogen-free, dedicated facility. They were handled following protocols approved by the Institutional Animal Care and Use Committee, National Defense Medical Center, Taiwan. General anesthesia was applied by intraperitoneal injection of 0.05 mg/kg atropine and 1.1 mL/100 g mixed titetamme and zolazepam (Zoletil 50, Virbac, Carros, French) and 0.05 mL/100 g xylazine hydrochloride (Rompun, Bayer, Leverkusen, Germany). Then, an incision was made on the calvaria region with a scalpel, a full-thickness flap was elevated, and the parietal bones were aseptically exposed. Two circular defects with dimensions of 6 mm × 1.8 mm were surgically created using a trephine (Komet, Hannover, Germany) powered by an electric motor under syringe irrigation. During the surgery, the scaffolds were chosen randomly from four groups, and all were precisely placed within the calvarial defects. The animals were sacrificed using carbon dioxide inhalation. The skulls were isolated and fixed in 4% paraformaldehyde on weeks 4 and 8. The samples were prepared for further investigation with dental radiography, μCT, and histological evaluation.

### 2.6 Dental Radiography

All groups were examined by digital radiography using a computerized imaging system to determine the extent of bone formation (Asahi Xspot; Asahi Roentgen Ind. Co., Ltd., Kyoto, Japan). The X-ray tube was operated at a distance of 2 cm from the source to the sensor, at 70 kV, with a current of 6 mA for 0.12 s. The image management system was used for image processing (INFINITT Dental PACS image system, INFINITT North America Inc., Phillipsburg, New Jersey, USA). Measurements in the mineralization areas of the central area were conducted using ImageJ (National Institutes of Health, Bethesda, Maryland, USA) and considered as the areas of new bone formation.

### 2.7 Micro-Computerized Tomography (Micro-CT)

Specimens were evaluated using micro-CT imaging with a high-resolution scanner (Skyscan1076, SkyScan, Aartselaar, Belgium). The tube was operated at 50 kVp accelerated potential, 18 μm image pixel size, 200 μA beam current for 460 ms, and a 0.8° rotation step. Medical image processing software was used for data collection and reconstruction (MiiL 3D, Visualization and Interactive Media Laboratory, National Center for High-performance Computing, Taiwan). The mineralized volume of bone (Bv) and the relative mineralized volume (Bv/Tv) in defects were measured.

### 2.8 Histological Analysis

For the processing of samples, the parietal bones were fixed in formalin overnight and treated with alcohol for dehydration of the samples before they were embedded into the resin.^51^ Eighty micrometer-thick sections were obtained from 64 calvaria specimens using a microtome (SP1600, Leica, Germany) and stained with toluidine blue stain. The slides were analyzed using a light microscope (DMI3000B, Leica, Germany).

### 2.9 Statistical Analysis

All data were analyzed using GraphPad Prism. The effect of calcification on the radiographic and histological results was compared among groups by the one-way ANOVA and post hoc analysis with Duncan’s test. Error bars indicate standard deviations in the graphs unless otherwise stated.

## 3 Results and Discussion

### 3.1 Fabrication of the Scaffolds

G1, G2, and G3 scaffolds were fabricated using 4PCLMA as a biomaterial. The detailed characterization of the material has been reported previously by our group.^1^ The control group, G4, was made of high molecular weight (50.000 g/mol), linear, thermoplastic PCL. Although commercially available thermoplastic PCL has been widely used for numerous biomedical applications for decades,^39−42^ there are a limited number of studies with thermoset PCL.^21,29,52,53^ Thermoset PCL mostly needs to be synthesized in house, and it has various advantages over thermoplastic PCL. Its molecular weight, degree of functionalization, and the number of arms can be designed for specific applications. It can be processed under mild operational conditions, and depending on the sample size, polymerization takes seconds to minutes. Polymerized substrates are autoclavable and have higher solvent resistance compared to thermoplastic PCL.

Materials with less than 2 nm and more than 50 nm pores are defined as microporous and macroporous, respectively, according to the International Union of Pure and Applied Chemistry (IUPAC). Materials with pore sizes between 2 and 50 nm are classified as mesoporous.^54^ However, there is an alternative classification introduced by Bose et al. in their well-accepted review article for, specifically, bone tissue engineering, where pores with a diameter of less than 20 μm and a pore diameter larger than 100 μm are categorized as microporous and macroporous, respectively. Accordingly, in this study, pores larger than 100 μm are defined as macropores and pores with a diameter of around 20 μm are named micropores.

Macro-photos and SEM images of the four groups of scaffolds are demonstrated in Figure 3A–F. The micropore sizes of G1 and G2 were distributed between 6 and 78 μm; the average pore size was calculated as 20 ± 11 μm. Also, micropores of the emulsion-templated scaffolds (G1 and G2) showed open-pore architecture, which is characterized by the presence of the windows between neighboring pores.^55^ The average macropore sizes of G2 and G3 were calculated as 0.84 ± 0.07 and 0.85 ± 0.03 mm, respectively, and no significant difference was found between the average pore sizes of the two groups. The macropores were designed as 0.84 mm in the template used for stereolithography. Thus, it can be revealed that scaffolds were fabricated with high accuracy (>98.8%) by our DMD setup. The average macropore size and the size of the struts of the G4 scaffolds were calculated as 0.90 ± 0.02 and 0.34 ± 0.03 mm, respectively.

Recently, hierarchical porous scaffolds have gained great attention.^56−59^ Multiscale porosity has been reported to be more advantageous for the regeneration of the bone compared to scaffolds with single-scale porosity.^60,61^ Fabrication of multiscale porous scaffolds using various techniques has been reported by other researchers.^8,58−60,62^ For example, Rustom et al. used a micro-robotic deposition system and porogen leaching for the fabrication of biphasic calcium phosphate with macro- and microsizes of >300 and <50 μm, respectively.^59^

The emulsion templating route is more commonly used to introduce microporosity into tissue engineering scaffolds (up to a couple of hundreds of micrometers). However, this technique can be easily combined with other techniques that can add macroporosity to create multiscale porous scaffolds following a multistep route. Recently, we have shown that emulsion templating can be combined with a syringe-based pneumatic extrusion system for the successful fabrication of hierarchically porous scaffolds.^1^ Also, recently, Paljevac et al. and Owen et al. reported the use of poly(dimethylsiloxane) (PDMS) beads and alginate beads, respectively, as a sacrificial molding material for the fabrication of multiscale porous materials.^6,63^ Owen et al.^64,65^ and Sherborne et al.^10^ combined emulsion templating with stereolithography for the fabrication of hierarchically porous scaffolds with macro- and microporosities.

Similarly, in this study, we first started to work on the fabrication of woodpile 4PCLMA PolyHIPE scaffolds. However, as the 4PCLMA HIPE composition contains a high percentage of solvent and during layer-by-layer fabrication, the solvent evaporated, and this caused destabilization of the emulsion, shrinkage of the scaffolds, distortion of the fabricated scaffold, and closed porosity. That is why we needed an alternative fabrication route to introduce macroporosity into our scaffolds while keeping the micropores open. To be able to ensure open-surface porosity, we obtained macropores by perforating the scaffolds, following the polymerization of the emulsion, and we successfully created macropores. Although this technique does not allow the fabrication of scaffolds with complex porosity, it has been shown as an alternative method for introducing second-grade pores practically.

*In vitro* biocompatibility of 4PCLMA PolyHIPE has been previously shown using human dermal fibroblasts.^29^ Its potential to be used as a bone tissue engineering scaffold also has been demonstrated using murine osteoblast/osteocyte-like cells (MLO-A5s) and human embryonic stem cell-derived mesenchymal progenitor cells (hES-MPs) for up to 4 weeks. 4PCLMA has been shown to be biocompatible, providing favorable morphology for attachment, proliferation, and infiltration of cells *in vitro*. We have shown the potential of microporous^30^ and multiscale porous (microporous and macroporous)^1^ 4PCLMA PolyHIPE-based scaffolds in bone regeneration *in vitro*; however, we have not compared their performance *in vivo* yet. Also, to date, there have been no studies reporting the *in vivo* performance of any PolyHIPE scaffolds for bone tissue engineering scaffolds. We only reported the performance of bone tissue engineering scaffolds in chick chorioallantoic membrane (CAM) assay.^1,30^ Thus, in this study, we investigated the bone regeneration potential of microporous, macroporous, and multiscale porous scaffolds in comparison *in vivo*. The bone regeneration abilities of the control and experimental groups were tested in the rat cranial defect model, and the experimental procedure is given in Figure 3. Scaffolds were implemented into calvarial defects, and an empty hole was used as a negative control. Scaffolds were isolated and analyzed at two time points: 4 and 8 weeks after implantation. X-ray, micro-CT analysis, and histological characterization have been carried out for the evaluation results.

### 3.2 *In Vivo* Study

#### 3.2.1 Radiography and Micro-CT

**Figure 4:**
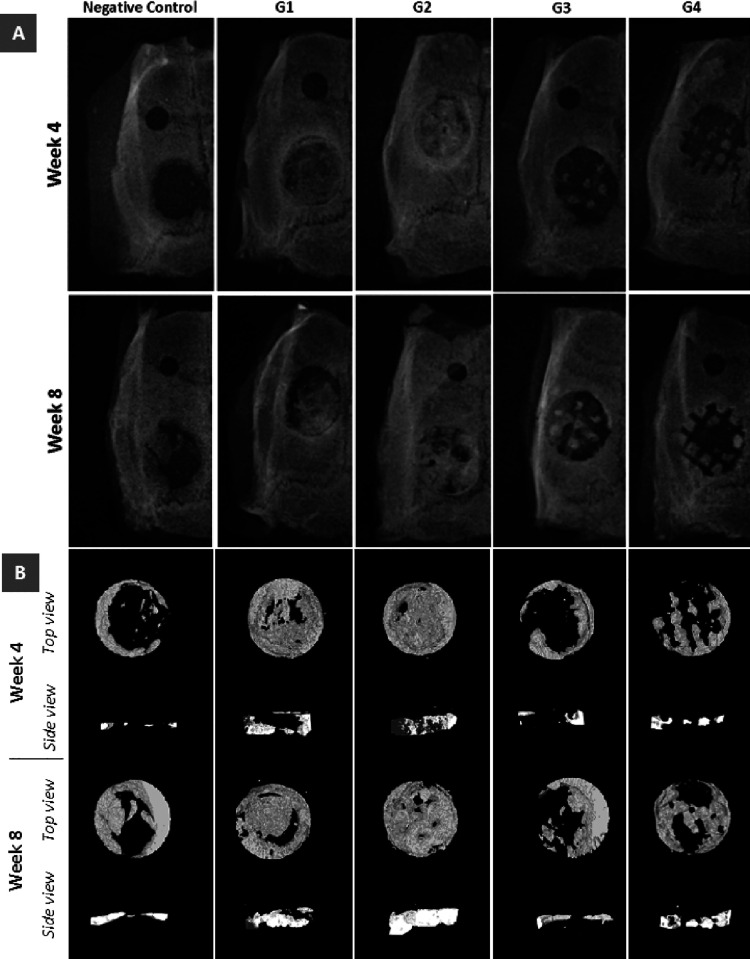
(A) Radiological analysis and (B) reconstructed micro-CT images at 4 and 8 weeks after implantation of negative control, G1, G2, G3, and G4 in rat calvarial defects.

**Figure 5:**
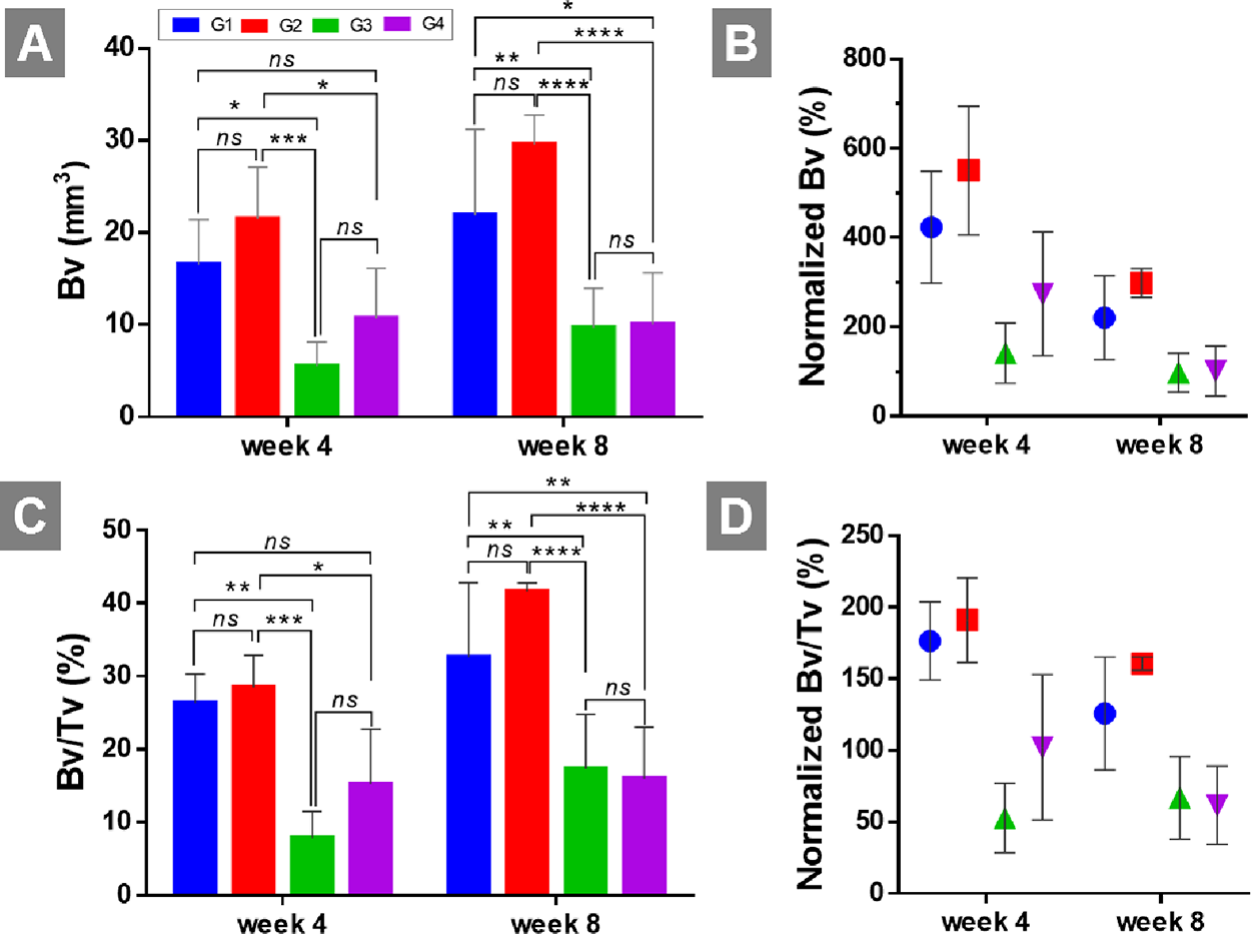
Quantitative data of μCT analysis. (A) Mineralized bone volume (Bv) and (B) normalized Bv (%) against control (negative control is assumed to have 100% Bv). (C) Relative bone volume:bone volume/tissue volume ratio (Bv/Tv) and (D) normalized Bv/Tv (%) against control (negative control is assumed to have 100% Bv/Tv) of the four groups at weeks 4 and 8 (****p < 0.0001, ***p < 0.001, **p < 0.01, and *p < 0.05).

For evaluation of *in vivo* performance of four groups of scaffolds, first, dental radiography and micro-CT were applied 4 and 8 weeks after implantation. Figure 4B shows the reconstructed specimens from the top and cross-sectional views. Figure 4A,B clearly shows that G1 and G2 showed better bone formation than G3 and G4. Reconstructed models show that, particularly, G2 exhibited better performance in terms of regeneration of bone in the defect hole. Figure 4A shows that due to a lack of microporosity, new mineralized bone formation was limited with macropores in G3 and G4. An empty hole (control) shows very limited regeneration when compared to G1 and G2, and that is mostly on the edges of the defect at two time points. However, on week 8, CT images and the normalized quantification graphs show that there is less bone ingrowth in G3 and G4 compared with the control (Figures 4 and 5). This is probably due to the absence of micropores in G3 and G4 and the scaffolds having bulk materials that limit space for tissue formation.

At week 4, bone volumes were measured as 16.5 ± 4.9, 21.5 ± 5.7, 5.5 ± 2.6, and 10.7 ± 5.4 mm^3^ for G1, G2, G3, and G4, respectively (Figure 5). G2 exhibited statistically significantly higher bone regeneration compared to G3 and G4 but not G1. There was no statistical significance between Bv values of G1–G2 and G3–G4. At week 8, the measured Bv value of G2 is around 3-fold that of G3 and G4. Similarly, although G2 has higher bone volume compared to G1, there was no statistical significance between them. While Bv of G1 was significantly higher than that of G3 but not G4 at week 4, Bv of G1 was significantly higher than that of both G3 and G4 at week 8. Bv/Tv graphs showed a similar trend with Bv graphs as all the groups have similar Tv values.

Both images and the quantification results showed that multiscale porous scaffolds (G2) that include both micro- and macropores resulted in higher bone regeneration in the defect area compared to macroporous scaffolds. When one-grade porous scaffolds were compared, microporous scaffolds (G1) have higher mineralized Bv than macroporous scaffolds (G3 and G4). There was no statistical difference between Bv values of G3 and G4 at any time point.

#### 3.2.2 Histological Evaluation

**Figure 6:**
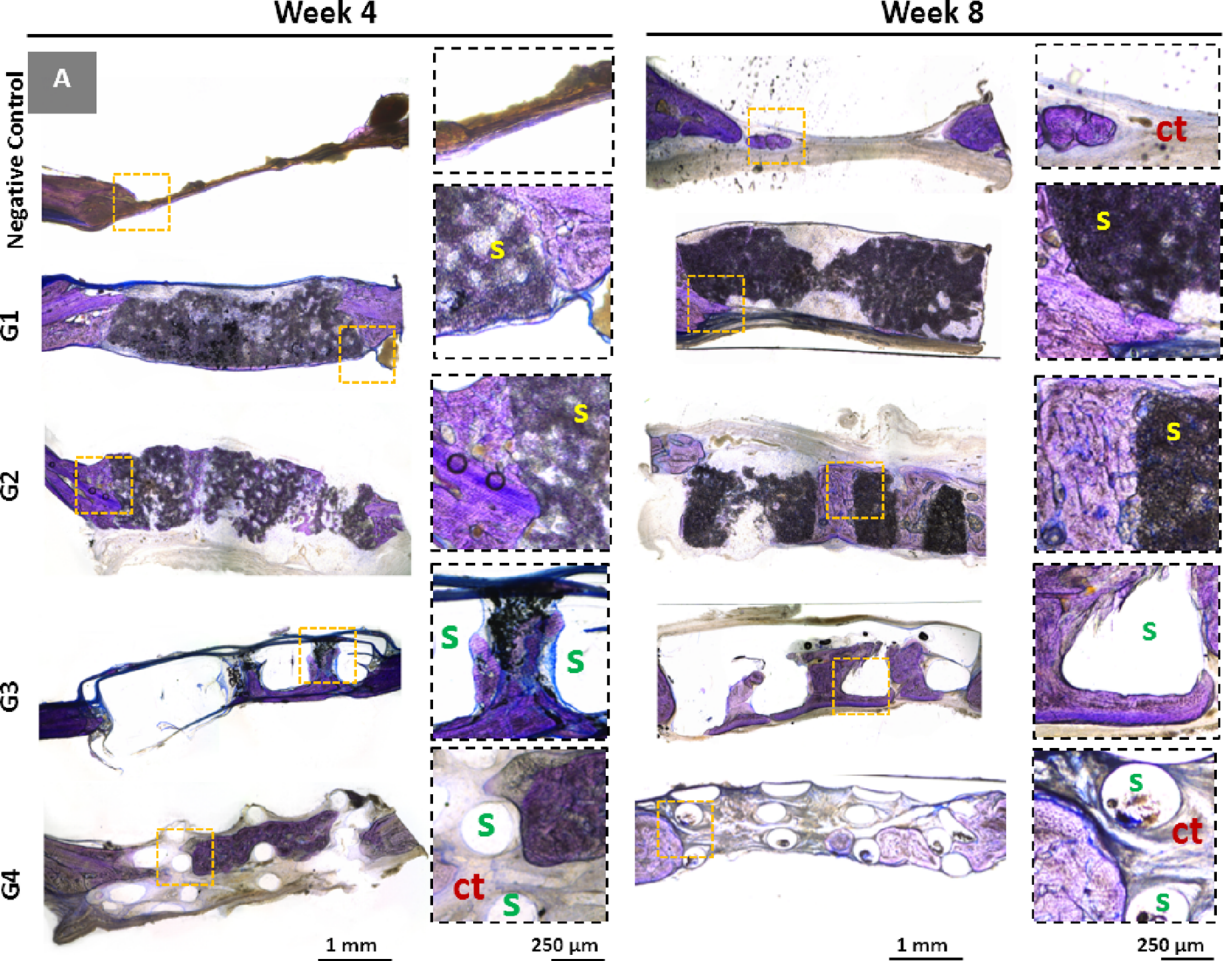
Histological results of rat calvarial defects 4 and 8 weeks after implantation. Toluidine blue staining: dark-blue staining indicates mineralized bone; connective tissue and unmineralized osteoid stain pale blue (s: scaffold; ct: connective tissue).

Histological analyses were conducted to investigate tissue infiltration into the scaffolds (Figure 6). Toluidine blue staining stains mineralized bone and connective tissue with dark blue and pale blue colors, respectively. There was not any significant tissue regeneration in the sham group, neither at the end of week 4 nor 8 weeks post implantation. Macropores of G3 and G4 can clearly be seen from the histological images. While there is a significant amount of infiltration into macropores due to the high volume of nonporous material, tissue regeneration stays limited in those regions.

Habibovic et al.^100^ implanted a biphasic calcium phosphate and hydroxyapatite (HA) scaffold with different microenvironments in the back muscles of Dutch milk goats, and they reported that micropores within macropore walls are crucial to increasing the osteoinductivity of the material. Conversely, Dang et al. fabricated PCL-based macroporous scaffolds using FDM and multiscale porous scaffolds by combining FDM and porogen leaching techniques, and the bone regeneration capacities of these scaffolds were tested in the rat calvarial defect model.^66^ They reported a similar level of bone formation in both groups of scaffolds.

Last, in G4, which is made of thermoplastic PCL, connective tissue infiltration is more apparent than in mineralized bone. On the contrary, a greater extent of tissue infiltration can be clearly seen in G1 and G2 as they have a higher surface area to accommodate the new tissue in the micropores.

## 4 Conclusions

In this study, we aimed to (i) test the *in vivo* performance of the microporous 4PCLMA PolyHIPE scaffold, (ii) compare the bone regeneration potential of micro/macro/multiscale porous 4PCLMA-based scaffolds, and finally, (iii) compare their performances with the thermoplastic PCL-based scaffold. We revealed that microporous 4PCLMA-based PolyHIPE scaffolds support new bone formation. When one-grade porous scaffolds were compared, microporous scaffolds showed better performance than macroporous scaffolds in terms of mineralized bone volume and tissue regeneration. Taken together, the results reported in this study demonstrated the potential application of especially multiscale 4PCLMA PolyHIPE scaffolds as a promising material for bone regeneration.
